# In Situ Miniaturised Solid Phase Extraction (m-SPE) for Organic Pollutants in Seawater Samples

**DOI:** 10.1155/2018/7437031

**Published:** 2018-04-02

**Authors:** B. Abaroa-Pérez, G. Sánchez-Almeida, J. J. Hernández-Brito, D. Vega-Moreno

**Affiliations:** ^1^Plataforma Oceánica de Canarias (PLOCAN), Las Palmas, Spain; ^2^Chemistry Department, Universidad de Las Palmas de G.C (ULPGC), Las Palmas, Spain

## Abstract

Solid phase extraction (SPE) is a consolidated technique for determining pollutants in seawater samples. The current tendency is to miniaturise systems that extract and determine pollutants in the environment, reducing the use of organic solvents, while maintaining the quality in the extraction and preconcentration. On the other hand, there is a need to develop new extraction systems that can be fitted to in situ continual monitoring buoys, especially for the marine environment. This work has developed a first model of a low-pressure micro-SPE (m-SPE) for persistent organic pollutants (POPs) that can be simply applied to in situ monitoring in the marine environment. This system reduces the volumes of sample and solvents required in the laboratory in comparison with conventional SPE. In the future, it could be used in automated or robotic systems in marine technologies such as marine gliders and oceanographic buoys. This system has been optimised and validated to determine polycyclic aromatic hydrocarbons (PAH) in seawater samples, but it could also be applied to other kinds of persistent organic pollutants (POPs) and emerging pollutants.

## 1. Introduction

Interest in controlling and monitoring different kinds of organic pollutants in marine environments has grown [1–4], due to the harm they can do to the marine environment and human health [5]. One example of these are polycyclic aromatic hydrocarbons (PAHs) that are considered as priority pollutants by the European Union (EU) and the Environmental Protection Agency (EPA) because they are carcinogenic and they can genetically mutate [3, 5–8] and what is more, these compounds could activate oxidative stress of DNA, hence damaging metabolic activation and the generation of reactive kinds of oxygen [9, 10] making the extraction, preconcentration, and determination of these compounds in the environment very important [11, 12]. PAHs are ubiquitous pollutants in the environment, with special importance in seawater [4, 11, 13, 14], sediments [15], plankton, and filtering organisms [5, 10, 16].

The concentration of PAHs in seawater is normally in the range of 0.05 to 0.25 *µ*g·L^−1^ [6, 17], due to their low solubility in water [1, 5, 18, 19]. A high concentration generally indicates PAH pollution of recent anthropogenic origin [6]. Over time, these compounds tend to accumulate in solid matrixes like sediment and marine plastic, with a strong tendency to bioaccumulate [16]. That is why new analytical methods are required that allow them to be monitored in situ while maintaining current levels of sensitivity and selectivity [6, 11, 20, 21].

The most commonly used techniques for determining PAHs are gas chromatography with mass spectrometry (GC-MS) [1, 2, 4] and high-pressure liquid chromatography with ultraviolet-visible detector or diode array ultra-violet-visible detector and fluorescence detector [11, 22–25]. In order to enhance the sensitivity and selectivity of the analyses, a first stage of extraction, purification, and preconcentration is required [5].

There are several preconcentration techniques for organic pollutants in liquid matrixes, such as liquid-liquid extraction (LLE), supercritical fluid extraction (SFE), and solid phase extraction (SPE) [5, 26, 27].

Solid phase extraction (SPE) is currently a highly consolidated technique for extracting pollutants from liquid samples [11, 28]. SPE gives high recoveries with a low consumption of organic solvents and high preconcentrations if volumes of water of around 1 litre are filtered. SPE has been widely used for extracting hydrocarbons and other persistent pollutants from seawater and other kinds of marine samples [1, 3, 4, 12, 29]. However, these laboratory studies require large volumes of seawater that are processed in laboratories, rather than directly at the place where the sampling is done, entailing the transport and storage of the samples, which makes the sampling operation more difficult [14]. This explains the special interest in miniaturising the extraction and its application in situ, to facilitate enormously the logistics of sampling, and also opening up the possibility of future automation.

There are other systems that have attempted to miniaturise extraction, like solid phase microextraction (SPME) [5, 30–32] or fabric phase sorptive extraction (FPSE) [33], which combine extraction and preconcentration in a single step [6, 17, 34]. The disadvantage of SPME is that it is not a very robust or reproducible system, and it is very difficult to handle in situ in the marine environment. FPSE is a new technique that has yet to be tested on marine samples.

There are very few studies that consider monitoring the effects of a polycyclic aromatic hydrocarbon spill; most have been done with biomarkers, without any analytical quantification [16]. Portable systems are required to facilitate the task and reduce time and material, which allow a study to be conducted in situ.

The m-SPE presented here has the advantage of its simplicity, low cost, and ease of installation in the place the sampling is to be conducted (in situ). This method has been validated for extracting and preconcentrating PAHs in seawater.

## 2. Materials and Methods

### 2.1. Developing an m-SPE System

**Figure 1:**
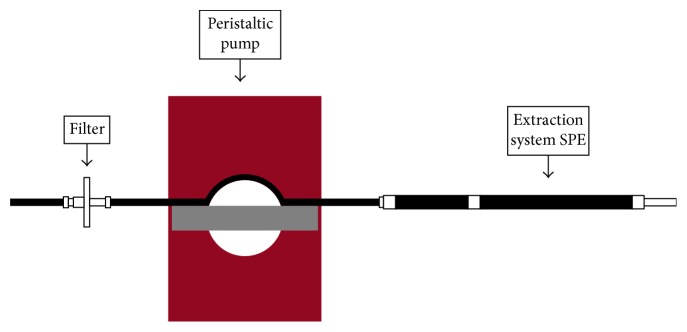
Miniaturised solid phase extraction system (m-SPE).

The miniaturised solid phase extraction system in question is shown in Figure 1 included an ISMATEC peristaltic pump, model: ISM 846 (60 rpm, dimensions 125 × 88 × 135 mm), with SKALAR connectors, model: 3091 with a theoretical flow rate of 0.14 ml·s^−1^. Behind the sample reservoir, there is a fibre-glass Whatman GF/C filter (porosity of 1.2 *µ*m) to eliminate possible solids from the seawater that could interfere with the analysis. The peristaltic pump pumps water up to the miniaturised SPE cartridge, which consists of a TYGON tube (inert, SC0359) with a diameter of 4.8 mm filled with the appropriate solid SPE sorbent for each analysis.

### 2.2. Chemical Reagents

The PAHs studied were fluoranthene, chrysene, benzo(b)fluoranthene, dibenzo(a,h)anthracene (Sigma-Aldrich®), and benzo(a)pyrene (Supelco®). The initial individual standard was dissolved in HPLC-grade acetonitrile (Panreac®).

A mixture of the 6 PAHs was prepared at a concentration of 10 mg·L^−1^ in methanol to study the recovery rates (LiChrosolv® Reag. Ph Eur Methanol gradient grade for liquid chromatography, Merck®). The seawater samples to be analysed are enriched with this mixture to validate the m-SPE system. In this case, 1 L of prefiltered seawater (Whatman GF/C glass-fibre filter, porosity of 1.2 *µ*m) was contaminated to eliminate any possible solutes that could interfere in the analysis. The concentration of seawater used to optimise the system was 0.2 *µ*g·L^−1^.

### 2.3. Solid Phase Extraction Procedure (m-SPE)

Miniaturising the SPE system is based on manufacturing sorbent cartridges that can be coupled to a peristaltic pump (avoiding the traditional vacuum pump). These cartridges were prepared with 0.3 g of Envi-18 (Supelco) silica gel, placed inside a tube with an internal diameter of 4.8 mm and 6 cm long. There is an IDEX 5 mm ISM560 joint at each end and a piece of polyethylene frit (Supelco) with a porosity of 20 *µ*m on the inside of each joint.

Samples of one litre of seawater with 0.2 *µ*g·L^−1^ of each of the six PAHs analysed were used to optimise extraction. After sampling, 50 mL of Milli-Q water is added through the system and it is left to dry, hence minimising the amount of water present before extraction. The presence of water can trigger a lower recovery and low reproducibility [28]. Finally, the pollutants are disorbed with methanol, and the first mL of extract is collected for analysis by HPLC with fluorescence detector.

### 2.4. PAH Analysis by High-Pressure Liquid Chromatography (HPLC) with Fluorescence Detector

The analysis of the samples was conducted in Varian® 230, fitted with a ProStar 3012 binary pump, which requires up to three entry lines of solvent and a ProStar Varian 410 self-sampler. The analytes of interest are put through a ProStar 363 fluorescence detector. The valve in the column is a 500-LC, with a Microsorb–MV 100-5 C18 ODS 150 × 4.6 mm × 1/4″ column.

The chromatographic column was kept at 30°C throughout the HPLC process to prevent variability due to environmental conditions. Consideration was given to the excitation and emission wavelengths of each of the PAHs to be analysed [35], and the range of an excitation length of 260 nm and an emission length of 440 nm was determined for the fluorescence detector, which enables the entire spectrum of the different PAHs to be seen.

The work was done on a gradient with a mobile phase A, methanol : water, in a proportion of 80 : 20, and a mobile phase B of 100% methanol. The method was applied on a gradient, lasting 18 minutes. It starts with 100% A, and then progressively increases the proportion of B until this reaches 100% B for 14 minutes. The last 4 minutes are to reestablish the initial conditions, ending with 100% A after 18 minutes.

## 3. Results and Discussion

### 3.1. Optimising the Miniaturised Solid Phase Extraction System (m-SPE)

In order to study the best SPE sorbent, extractions were made under different conditions for a reference sample (seawater with 0.2 *µ*g·L^−1^ for the 5 PAHs studied). The main conditions studied were the kind of sorbent used and how much of it, in grams.

#### 3.1.1. Comparison of Different Solid Sorbents

The different kinds of cartridges to be used in the SPE are classified in accordance with the analytes of interest [17]. In this work, the right adsorbent for studying PAHs in seawater was assessed. Different brands and models of SPE cartridges were used for the filling. The cartridges used were the Supelco Envi-18, Thermo® scientific Hypersep SCX, and the Interchim® Upti-Clean.

The same procedure was used with each kind of cartridge. It was run three times with 1 litre of prefiltered seawater contaminated with 0.2 *µ*g·L^−1^. The pollutants are disorbed with 1 mL of methanol, and then they are analysed in the HPLC. Two different amounts were used to determine the best sorbent, 0.4 g in the first, and then the two best results were studied with 0.3 g for each kind of sorbent.

**Figure 2:**
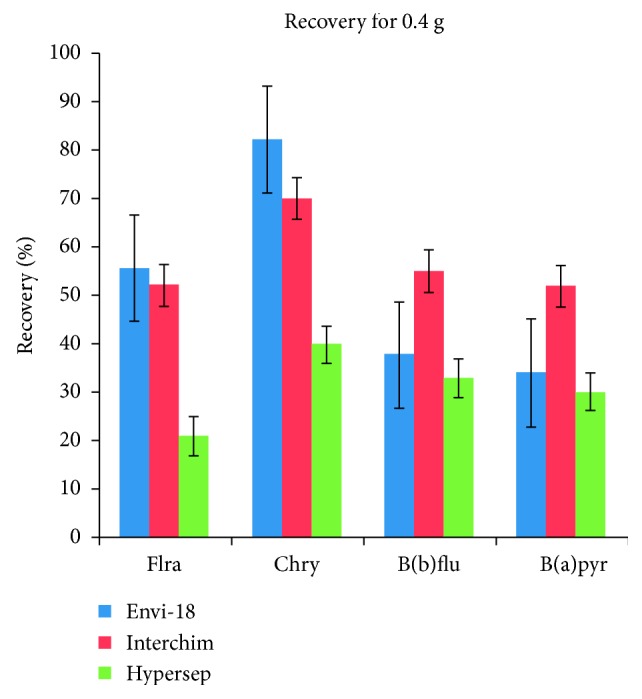
Comparison of different sorbents using a dosage of 0.4 grams: fluoranthene (Flra), chrysene (Chry), benzo(b)fluoranthene (B(b)flu), and benzo(a)pyrene (B(a)pyr).

**Figure 3:**
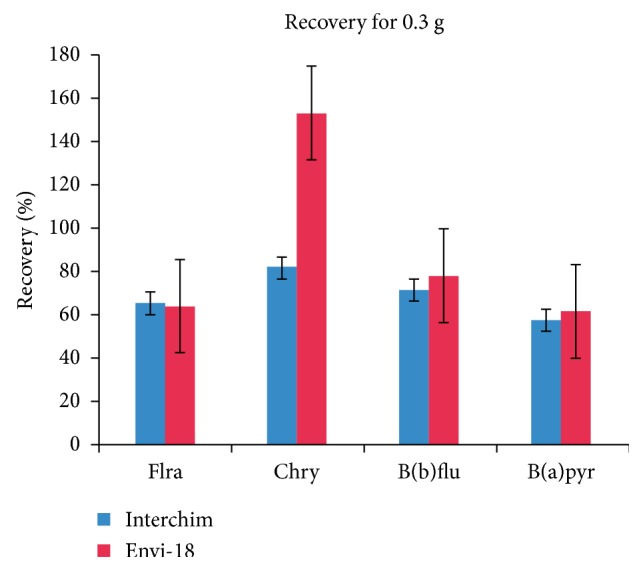
Comparison of the best two sorbents using a dosage of 0.3 grams: fluoranthene (Flra), chrysene (Chry), benzo(b)fluoranthene (B(b)flu), and benzo(a)pyrene (B(a)pyr).

Thermo scientific Hypersep SCX is the sorbent that presents the lowest recovery percentage with 0.4 g of sorbent (Figure 2), which is why it was eliminated from the next study, using a smaller amount of sorbent.  Figure 3 shows that the recovery percentage for the Supelco Envi-18 sorbent shows better results than the Interchim Upti-Clean.

#### 3.1.2. Comparison of Different Amounts of Sorbent

**Figure 4:**
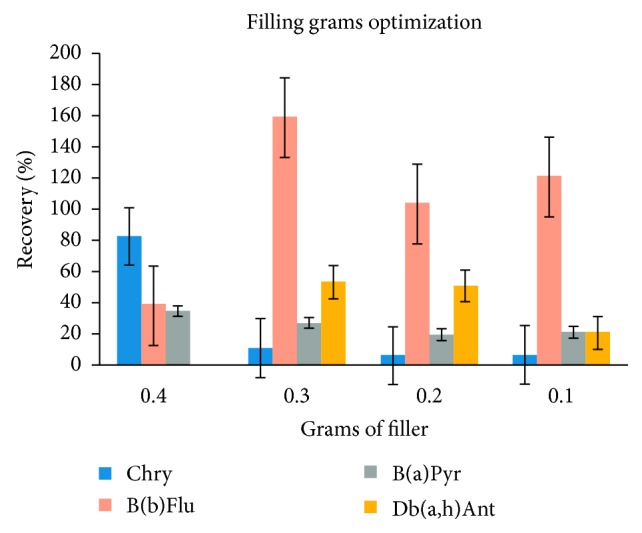
Optimising the amount of sorbent (in grams): chrysene (Chry), benzo(b)fluoranthene (B(b)Flu), benzo(a)pyrene (B(a)pyr), and dibenzo(a,h)anthracene (Db(a,h)ant).

Once the kind of sorbent to be used was optimised, the results for the different doses of sorbent (in grams) were analysed. The three different doses of Envi-18 silica gel in the extraction cartridge were compared: 0.3, 0.2, and 0.1 grams. One litre of prefiltered seawater was contaminated with 0.2 *µ*g·L^−1^, and it was run through the extraction system with each of the dosages. The results are shown in Figure 4, showing that the concentrations are higher in the m-SPE cartridge with the highest dose of silica gel.

### 3.2. Analytical Reproducibility and Application

The analytical method proposed was assessed under the optimum conditions mentioned above, giving a relative standard deviation (RSD) for an extraction of a sample of 1 litre at 0.2 *µ*g·L^−1^ of the mixture of PAHs.

The RSDs (%) obtained are shown in Table 1 and are around 4.21 and 10.27% for each PAH analysed. The limit of detection study gave very low results, as did the limit of quantification.

The applicability of the method was assessed using real samples in situ (without spike) collected from different places on the island of Gran Canaria, Canary Islands, Spain: two points in the east of the island, (Port of Taliarte and Port of Salinetas) and one further north, where the largest port of the island is located (Port of Las Palmas de Gran Canaria). In each case, 1 litre of surface seawater was collected per sample, with three samples taken at each point.

**Figure 5:**
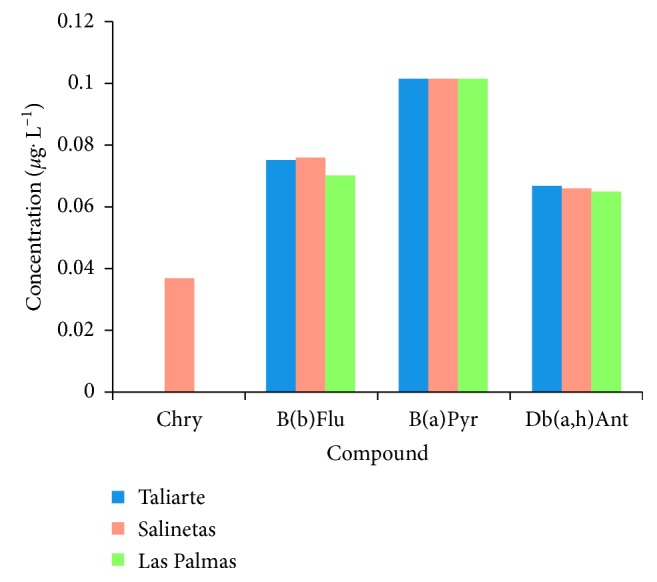
Results of applying the method to real, uncontaminated samples at different points of Gran Canaria, Canary Islands, Spain.

The volume taken for each sample was measured by collection time based on its flow rate, giving a total of 119 minutes per sample. The results of the real samples analysed are shown in Figure 5. The PAHs with the highest molecular weight are more hydrophobic than the PAHs with low molecular weight [36–38] (the chrysene is the most soluble, and dibenzo(a,h)anthracene is the least soluble), and this is reflected in the concentrations obtained, as they always have a lower presence in water.

The concentrations in the different areas of Gran Canaria varied significantly in several cases below the limit of detection. These figures do not exceed the limits permitted by the legislation in effect [39].

## 4. Conclusions

The m-SPE in this study was developed to extract PAHs for seawater samples and presented a robust PAH extraction capacity, even for very low concentrations in liquid samples, thus, guaranteeing that the method is able to detect and quantify concentrations below the limits set by law. This means that it is a reliable method for assessing concentrations of directive 2008/105/CE [39].

The reproducibility of the method could be improved by using pollutant separation with gas chromatography with mass spectroscopy (GC-MS) that offers improvements against high-pressure liquid chromatography (HPLC) [2, 9, 21, 40], showing greater sensitivity in the analysis of these pollutants.

These results show the feasibility of the in situ extraction process using miniaturised, solid phase extraction. The methodology developed in this study is simple, fast, easy, and allows for in situ sampling. It is also a sustainable methodology because the use of organic solvents is minimal. It represents the first step towards automating extraction in ports and coastal areas such that monitoring can be conducted more frequently without the need for frequent sampling.

The potential of this system is that it can be fitted to submarine vehicles and oceanic buoys, allowing for continual, efficient, and low-cost monitoring of the quality of the ocean.

## Figures and Tables

**Table 1:** Results of the relative standard deviation (% RSD), limit of detection (LOD), and limit of quantification (LOQ).

Compound	Abbreviation	% RSD	LOD (ng·L^−1^)	LOQ (ng·L^−1^)
Chrysene	Chry	4.21	0.22	0.72
Benzo(b)fluorantene	B(b)Flu	9.57	0.20	0.67
Benzo(a)pyrene	B(a)Pyr	10.27	0.30	1.00
Dibenzo(a,h)anthracene	Db(a,h)ant	9.88	0.02	0.06
